# Education of Law-Makers1Read at twenty-fourth regular meeting of the Missouri Valley Veterinary Association, St. Joseph, Mo., June 25, 1900.

**Published:** 1900-09

**Authors:** Joseph W. Parker

**Affiliations:** Kansas City, Mo.


### EDUCATION OF LAW-MAKERS.[1]

By Joseph W. Parker, D.V.S.,
KANSAS CITY, MO.

Modern politics appeals to intelligence. A bureau of publication is now an essential feature of every successful political campaign. It has been said that the education of a child should begin three generations before it is born. To paraphrase, it might be said that the education of a law-maker must begin with the people who elect him, or on whom he depends for further political successes. Whatever a large part of the people demand, their representatives must and will enact. Legislators are seldom far in advance of their constituency.

The concentrated primary aim of veterinarians relative to needed legislation, therefore, must be to inform the public by precept and example that the profession is one which should

[1] Read at twenty-fourth regular meeting of the Missouri Valley Veterinary Association, St. Joseph, Mo., June 25, 1900.

be regulated by law, that the demands for legislation in these matters arise from the needs of the public. In the practice of veterinary medicine the interests of the patron and of the general public are involved. The immense value of domestic animals and their products, and the intimate relations existing between diseases of the lower animals and of man, justify and demand not only that those who are permitted to practice shall be required to furnish evidence of their qualifications, but laws to effectually control the spread of infectious and contagious diseases and for the supervision of the food supply of the public.

It devolves upon regular veterinarians to be the leaders in the campaign of political agitation and education, as by their training they are especially fitted to be. The approaching biennial sessions of the Legislatures of Missouri, Kansas, and Iowa, and the State elections to be held next fall make this a peculiarly fitting time and occasion for renewal of activity on the part of veterinarians, and especially on the part of this Association. Whether we succeed at this time or not in securing the recognition at which we aim, we will be laying the foundations for success in the future. I take it that there is no question in the mind of anyone present whether the members of the Missouri Valley Veterinary Association will loyally co-operate to secure legislation next winter; it is only a question of how we shall approach the task before us. It is with the hope of exciting discussion which shall evolve a practical plan of action that the essayist has undertaken to present this subject before you.

The foundations of the campaign must be established in the minds of voters, broad and strong, as befits the growing importance of the subject. The public is to be reached in three ways, viz.: Through personal contact, from the speaker's platform, and through publications.

First in importance is personal influence. To have intimately known a man of high honor is to have an exalted opinion of man in general. The community that has been fortunate enough to have in its midst a qualified, honorable veterinarian has had its opinion of that profession elevated and broadened. No matter whether the doctor be eloquent or not, or whether he be able to write a convincing article in the most polished language, if he is one of nature's noblemen, honest, conscientious, diligent, and thorough in business, and courteous in his business and social intercourse, he is elevating his profession in the minds of his patrons and fellow-citizens. He shows forth, in a very practical and convincing way, that his personal interests are in harmony with the interests of his patrons and with the public good.

While it is essential that our calling shall be regarded favorably as a learned and highly useful one, our demands for legislation must be for those things which are to the interest of the public. The profession is dignified by the earnestness with which it seeks after the public good, not primarily selfish interests. Whatever degree of respect the title "veterinarian" commands is the result of the silent teaching of a very limited number of veterinarians (for in these States we are few and far between), and in spite of the disrepute resulting from itinerary practice of hundreds of quacks. By maintaining among the profession a high standard of personal and professional ethics, thus increasing the usefulness of the calling, favorable opinions must be won of the public before they may be expected to listen to veterinarians in matters of legislation.

For reaching people interested I believe that farmers' institutes offer opportunities promising good results. If arrangements can be made with the promoters of farmers' institutes to place a veterinarian on each programme whenever practicable, doubtless competent men can be found who, for their interest in the progress of the public and the advancement of veterinary science, will prepare papers or addresses on subjects that interest both farmers and veterinarians. At suitable times exhibits might be made a feature of the veterinary talk. To effect this, co-operation of veterinarians is essential, for no one man can afford to take from his time and business to attend all farmers' institutes unless salaried for that purpose. Stockmen's meetings also afford excellent opportunities for the education of the public, and a veterinarian should be on every programme. As men making some pretensions to learning, veterinarians must identify themselves with progress in all matters related to their work.

We are fond of referring to the broad field of usefulness of our profession, but should remember that it is useful to others only as we make it so, and it rewards us according to our deserving. Granted that the veterinary profession has already won in these States the respect of those who have influence in our legislative bodies, and that our demands are for those laws which would be for the benefit of the public, we may hope to accomplish something at the ensuing election and with the State Legislatures.

Newspapers may also be used to advantage at times. In agitations for pure food-supply, control of infectious and contagious diseases, sanitation, and kindred topics, the columns of the secular press will certainly be utilized, and their articles should not be inspired by ignorance of the true merits of the questions, or by malice of those whose interests are opposed to the interests of the public.

Legislators are to be influenced by the same considerations which influence the opinions of others, by public sentiment, by appeal to their personal ambition and vanity, and by lobbying. I believe that the average statesman (pardon the misapplication of the word) oftener asks himself whether this or that official act will improve his chances of further political preferment than he concerns himself with the public good. When the interests of his constituency and his own political prospects are in harmony, well and good; but if there is apparent conflict, the interests of the public are usually sacrificed.

As veterinarians we must, therefore, become practical politicians and lobbyists; must be known as local writers for the secular press, and as speakers on all suitable occasions. We must co-operate with mind, money, and motive; and, above all and including all these, must be imbued with professional *esprit du corps*, and actuated by altruistic motives.

---

THE Minnesota State University, in which Dr. Reynolds holds full professorship, ranks second in point of attendance among the colleges and universities of America. The enrollment last year was about 3300. This includes no preparatory students, as this University has long since done away with preparatory courses, and students can only enter when well prepared for advanced genuine collegiate work. Harvard ranks first, University of Minnesota second, and University of Michigan third. This note emphasizes some remarks made at the last banquet of the A. V. M. A. concerning the marvellous growth of Western universities. This growth seems to be true not only in point of attendance, but in the way of magnificent buildings, splendid laboratory equipment, and well-paid faculties.

